# Increased Excitatory Synaptic Transmission of Dentate Granule Neurons in Mice Lacking PSD-95-Interacting Adhesion Molecule Neph2/Kirrel3 during the Early Postnatal Period

**DOI:** 10.3389/fnmol.2017.00081

**Published:** 2017-03-22

**Authors:** Junyeop D. Roh, Su-Yeon Choi, Yi Sul Cho, Tae-Yong Choi, Jong-Sil Park, Tyler Cutforth, Woosuk Chung, Hanwool Park, Dongsoo Lee, Myeong-Heui Kim, Yeunkum Lee, Seojung Mo, Jeong-Seop Rhee, Hyun Kim, Jaewon Ko, Se-Young Choi, Yong Chul Bae, Kang Shen, Eunjoon Kim, Kihoon Han

**Affiliations:** ^1^Department of Biological Sciences, Korea Advanced Institute of Science and Technology (KAIST)Daejeon, South Korea; ^2^Center for Synaptic Brain Dysfunctions, Institute for Basic Science (IBS)Daejeon, South Korea; ^3^Department of Anatomy and Neurobiology, School of Dentistry, Kyungpook National UniversityDaegu, South Korea; ^4^Department of Physiology, Dental Research Institute, Seoul National University School of DentistrySeoul, South Korea; ^5^Department of Neurology, Columbia University Medical CenterNew York, NY, USA; ^6^Department of Anesthesiology and Pain Medicine, College of Medicine, Chungnam National UniversityDaejeon, South Korea; ^7^Department of Neuroscience, College of Medicine, Korea UniversitySeoul, South Korea; ^8^Department of Anatomy, College of Medicine, Korea UniversitySeoul, South Korea; ^9^Department of Molecular Neurobiology, Max Planck Institute of Experimental MedicineGöttingen, Germany; ^10^Department of Brain and Cognitive Sciences, Daegu Gyeongbuk Institute of Science and Technology (DGIST)Daegu, South Korea; ^11^Department of Biology, Howard Hughes Medical Institute, Stanford UniversityStanford, CA, USA

**Keywords:** Neph2, Kirrel3, PSD-95, excitatory synapse, dentate granule neuron

## Abstract

Copy number variants and point mutations of *NEPH2* (also called *KIRREL3*) gene encoding an immunoglobulin (Ig) superfamily adhesion molecule have been linked to autism spectrum disorders, intellectual disability and neurocognitive delay associated with Jacobsen syndrome, but the physiological roles of Neph2 in the mammalian brain remain largely unknown. Neph2 is highly expressed in the dentate granule (DG) neurons of the hippocampus and is localized in both dendrites and axons. It was recently shown that Neph2 is required for the formation of mossy fiber filopodia, the axon terminal structure of DG neurons forming synapses with GABAergic neurons of CA3. In contrast, however, it is unknown whether Neph2 also has any roles in the postsynaptic compartments of DG neurons. We here report that, through its C-terminal PDZ domain-binding motif, Neph2 directly interacts with postsynaptic density (PSD)-95, an abundant excitatory postsynaptic scaffolding protein. Moreover, Neph2 protein is detected in the brain PSD fraction and interacts with PSD-95 in synaptosomal lysates. Functionally, loss of Neph2 in mice leads to age-specific defects in the synaptic connectivity of DG neurons. Specifically, *Neph2^−/−^* mice show significantly increased spontaneous excitatory synaptic events in DG neurons at postnatal week 2 when the endogenous Neph2 protein expression peaks, but show normal excitatory synaptic transmission at postnatal week 3. The evoked excitatory synaptic transmission and synaptic plasticity of medial perforant pathway (MPP)-DG synapses are also normal in *Neph2^−/−^* mice at postnatal week 3, further confirming the age-specific synaptic defects. Together, our results provide some evidence for the postsynaptic function of Neph2 in DG neurons during the early postnatal period, which might be implicated in neurodevelopmental and cognitive disorders caused by *NEPH2* mutations.

## Introduction

Synaptic adhesion molecules play diverse roles in synaptic development and function, including synapse specificity, formation, maturation and plasticity (Shen and Scheiffele, 2010; Tallafuss et al., 2010; Williams et al., 2010; Nam et al., 2011; Siddiqui and Craig, 2011; Yuzaki, 2011; Missler et al., 2012; Valnegri et al., 2012; Takahashi and Craig, 2013; Um and Ko, 2013; Yogev and Shen, 2014; de Wit and Ghosh, 2014, 2016; Han et al., 2016). Supporting their critical roles in normal brain function, many genes encoding synaptic adhesion molecules have been associated with multiple neurodevelopmental and neuropsychiatric disorders such as autism spectrum disorders, intellectual disability, schizophrenia and bipolar disorder (Südhof, 2008; Betancur et al., 2009; O’Dushlaine et al., 2011; Valnegri et al., 2012; Takahashi and Craig, 2013; Um and Ko, 2013).

Neph/Kirrel protein is a family of immunoglobulin (Ig) superfamily adhesion molecules (Sellin et al., 2003; Yogev and Shen, 2014) with three known members, Neph1/Kirrel1, Neph2/Kirrel3 and Neph3/Kirrel2, which were originally identified as junctional components of the kidney slit diaphragm (Donoviel et al., 2001; Sellin et al., 2003). Importantly, in case of *NEPH2/KIRREL3* gene in human chromosome 11q24.2, point mutations and chromosomal abnormalities were identified in autism spectrum disorders, intellectual disability and Jacobsen syndrome characterized by neurocognitive delay (Bhalla et al., 2008; Guerin et al., 2012; Michaelson et al., 2012; Talkowski et al., 2012), suggesting its important roles in brain.

The Neph2 protein contains five Ig-like domains in the extracellular region, followed by a single transmembrane domain (TM) and a cytoplasmic region that ends with a type I PDZ domain-binding motif. Neph2 shows homophilic interaction through its extracellular domain (Martin et al., 2015), and also interacts with other proteins such as MAP1B, MYO16, ATP1B1, SHMT2, UFC1 and CASK (Gerke et al., 2006; Bhalla et al., 2008; Liu et al., 2015). Among them, CASK is a presynaptic scaffolding protein implicated in X-linked brain malformation and intellectual disability (Hsueh, 2006; Najm et al., 2008) suggesting the potential roles of the Neph2 protein complex in synaptic development and function (Bhalla et al., 2008).

Functionally, SYG-1, the Kirrel ortholog in *Caenorhabditis elegans*, is expressed in HSNL motor neurons and determines the location of specific synapses by interacting with its ligand SYG-2 displayed on vulval epithelial cells (Shen and Bargmann, 2003; Shen et al., 2004). In the mouse brain, Neph2 proteins are expressed in diverse regions including the cortex, hippocampus, striatum, olfactory bulb and cerebellum (Gerke et al., 2006; Choi et al., 2015). Neph2 regulates axonal fasciculation and targeting in the olfactory and vomeronasal systems (Serizawa et al., 2006; Prince et al., 2013). In the hippocampus, Neph2 is highly expressed in dentate granule (DG) neurons and scattered GABAergic neurons of the hilus and CA3 (Choi et al., 2015; Martin et al., 2015). It was recently shown that Neph2, potentially through its extracellular homophilic interaction, is required for formation of mossy fiber filopodia, the axon terminal structure of DG neurons forming synapses with the GABAergic neurons of CA3 (Martin et al., 2015). Since the GABAergic neurons mediate feed-forward inhibition from DG to CA3 neurons, loss of mossy fiber filopodia in *Neph2^−/−^* mice is thought to over-active CA3 neurons.

Neph2 is also localized to neuronal dendrites and postsynaptic compartments (Gerke et al., 2006; Martin et al., 2015). However, in contrast to the above mentioned axonal and presynaptic functions of Neph2, its physiological roles in postsynaptic compartments remain unknown. In this study, we report that Neph2 directly interacts with postsynaptic density (PSD)-95, an abundant excitatory postsynaptic scaffolding protein, both *in vitro* and *in vivo*. Functionally, we found that *Neph2^−/−^* mice show significantly increased frequency of miniature excitatory postsynaptic currents (mEPSCs) in DG neurons at postnatal week 2 when endogenous Neph2 protein expression peaks, but it becomes normal at postnatal week 3. Our results provide a new insight into the synaptic roles of Neph2, which might be implicated in the disorders caused by *NEPH2* mutations.

## Materials and Methods

### cDNA Constructs

Full-length mouse Neph1 (aa 1–789) and mouse Neph3 (aa 1–700) cDNAs were amplified from brain cDNA libraries (Clontech), and human Neph2 (aa 1–778) cDNA was amplified from a KIAA clone (KIAA1867) obtained from the Kazusa DNA Research Institute. The full-length cDNAs were subcloned into GW1 (British Biotechnology). Full-length human Neph2 was subcloned into p3XFlag-N1. Partial cytoplasmic regions of Neph1 (aa 556–789), Neph2 (aa 720–778; WT, T776E and V778A) and Neph3 (aa 602–700) were subcloned into pBHA. Last seven amino acid residues of Neph1/2/3 (WT and V to A mutant) were subcloned into pGEX4T-1 (Amersham Biosciences). Full-length cytoplasmic region of Neph2 (aa 560–778; WT, Δ3 and T776E) was subcloned into p3XFlag-CMV-7.1 (Sigma-Aldrich).

### Antibodies

Neph2 antibodies generated by immunizing GST-fusion protein containing human Neph2 (aa 563–778; #1344 rabbit) and synthetic peptide mimicking the last 10 aa of human Neph2 (#1468 rabbit) have been described previously (Choi et al., 2015). GFP, GRIP2, PSD-93, PSD-95, SAP97, SAP102, synaptophysin and SynGAP antibodies have been described previously (Choi et al., 2005).

### Animals

*Neph2^−/−^* mice have been reported (Prince et al., 2013; Choi et al., 2015; Martin et al., 2015). *Neph2^−/−^* mice were maintained in the background of C57BL/6J, and all mice used in experiments were obtained by mating heterozygous mice. Mice were bred and maintained according to the Requirements of Animal Research at Korea Advanced Institute of Science and Technology (KAIST), and all procedures were approved by the Committee of Animal Research at KAIST (KA2012-19). Mice were fed *ad libitum* and 2–6 animals were housed in a cage under 12-h light-dark cycles. For some biochemical experiments, Sprague-Dawley rats (origin of Charles River Laboratory Inc.) were purchased and sacrificed on the day of arrival.

### Coimmunoprecipitation

Coimmunoprecipitation with rat brain lysates was performed as described previously (Choi et al., 2005; Han et al., 2009). The crude synaptosomal fraction of rat brains was solubilized with deoxycholic acid (DOC) buffer (50 mM Tris-HCl, 1% sodium DOC, pH 9.0), dialyzed against the binding/dialysis buffer (50 mM Tris-HCl, 0.1% Triton X-100, pH 7.4) and centrifuged. 50–100 μg of the supernatant was incubated with PSD-95, Neph2, or heat-denatured antibodies (negative control) for 2 h and then with protein A-Sepharose (Amersham Biosciences) for 2 h. The precipitates were analyzed by immunoblotting with indicated antibodies.

### Brain Fractionation

Subcellular and PSD rat brain fractions were prepared as described previously (Han et al., 2009). Briefly, rat brains were homogenized in buffered sucrose solution (0.32 M sucrose, 4 mM HEPES, 1 mM MgCl_2_, 0.5 mM CaCl_2_, pH 7.3) with freshly added protease inhibitors. This homogenate (fraction H) was centrifuged at 900 g for 10 min (the resulting pellet is P1). The resulting supernatant was centrifuged again at 12,000 g for 15 min (the supernatant after this is S2). The pellet was resuspended in buffered sucrose and centrifuged again at 13,000 g for 15 min (the resulting pellet is P2, crude synaptosome). The S2 fraction was centrifuged at 250,000 g for 2 h (the resulting supernatant is S3, and pellet is P3). The P2 fraction was resuspended in buffered sucrose and nine volume of water was added. After homogenization, the homogenate was centrifuged at 33,000 g for 20 min (the resulting pellet is LP1). The resulting supernatant was centrifuged at 250,000 g for 2 h (the resulting supernatant is LS2, and pellet is LP2). To obtain PSD fractions, the synaptosomal fraction was extracted with detergents, once with Triton X-100 (PSD I), twice with Triton X-100 (PSD II) and once with Triton X-100 and once with sarcosyl (PSD III), as described previously (Carlin et al., 1980; Cho et al., 1992).

### *In Situ* Hybridization

*In situ* hybridization was performed essentially as previously described (Kim et al., 2002). Hybridization probes specific for mouse Neph1/2/3 mRNAs were prepared using the following regions: nt 1861-2370 of Neph1 (NCBI accession #: AF480411), nt 2161-2694 of Neph2 (AY169782) and nt 2222-2770 of Neph3 (AK049284). Antisense riboprobes were generated using α-^35^S-UTP and the Riboprobe system (Promega).

### Neuron Culture, Transfection and Immunocytochemistry

DG neurons were prepared from postnatal day 4 rat brain and cultured as described previously (Jaworski et al., 1999). Cultured DG neurons at 8 days *in vitro* (DIV 8) were transfected using mammalian transfection kit (Invitrogen) and fixed at DIV 10 with 4% paraformaldehyde/sucrose, permeabilized with 0.2% Triton X-100, and incubated with primary and dye-conjugated secondary antibodies.

### Electrophysiology

For slice preparation, following isoflurane anesthetization WT or *Neph2^−/−^* mice, brains were removed and sliced in sagittal sections (300 μm) across the dorsal hippocampus. The sections were made in a (5% CO_2_) carbogen-bubbled, ice-cold sucrose CSF (sCSF) consisting of–in mM–212 sucrose, 25 NaHCO_3_, 5 KCl, 1.25 NaH_2_PO_4_, 10 D-glucose, 2 Na-pyruvate, 1.2 Na-ascorbate, 3.5 MgCl_2_, 0.5 CaCl_2_ using a vibratome (VT1220s, Leica). Slices were recovered at 32°C for 30 min in artificial CSF (aCSF) consisting of—in mM—125 NaCl, 25 NaHCO_3_, 2.5 KCl, 1.25 NaH_2_PO_4_, 10 D-glucose, 1.3 MgCl_2_, 2.5 CaCl_2_ and maintained thereafter at room temperature.

Recordings were made by using Multiclamp 700B amplifer and the Axopath 200B (Molecular Devices) under visual control with differential inference contrast illumination in an upright microscope (Nikon) and were filtered at 2 kHz and digitized at 10 kHz. Data were acquired via Clampex 10 (Molecular Devices) and analyzed by Clampfit 10 (Molecular Devices) or using custom macros written in Igor (Wavemetrics).

To record mEPSCs (P14–17, P20–22), hippocampal sections were perfused with aCSF containing 0.5 μM tetrodotoxin, 100 μM picrotoxin and the recording chamber was maintained at constant temperature (26.5°C–28°C) using a temperature controller (Warner).

The cells were voltage clamped at −70 mV. Recording pipettes pulled from borosilicate glass capillaries (Harvard Apparatus 1.5 mm OD) of 3–3.8 MΩ resistance were filled with a Cs gluconate-based internal solution of 280–290 mOsm (pH 7.3) containing–in mM–110 Cs gluconate, 8 NaCl, 10 TAEC, 20 HEPES, 5 Qx-314Cl, 4 Mg-ATP, 0.3 Na-GTP, 0.5 EGTA.

To minimize time-dependent fluctuation, the baseline was monitored for 5 min and mEPSCs were measured at the same time point after the establishment of whole-cell. Liquid junction potential correction was not taken into account. *Series access resistance (Ra)* was monitored (15–20 MΩ), and any data with Ra greater than 25 MΩ or greater than 20% change over the course of recording were discarded.

For input/output and paired-pulse ratio recordings, hippocampal sections were perfused with aCSF. Stimulating and recording pipettes were each filled with aCSF. For the input/output ratio, field excitatory postsynaptic potentials (fEPSPs) were monitored until a stable baseline of predetermined values was observed. Three sweeps per stimulus strength was recorded and averaged for each slice. PPR protocol was thereafter applied and recorded.

For theta-burst stimulation (TBS) long-term potentiation (LTP), hippocampal sections were perfused with aCSF. Stimulating and recording pipettes were each filled with aCSF. After a stable baseline of 20 min was observed and recorded, a TBS stimulus consisting of 10 stimulus trains delivered at 5 Hz with each train consisting of four pulses at 100 Hz was applied, and the responses were recorded for an hour.

## Results

### PDZ Interaction between Neph2 and PSD-95

**Figure 1:**
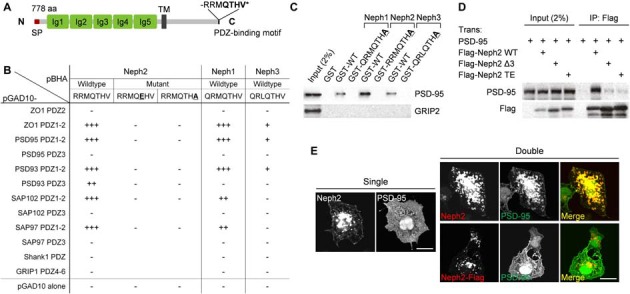
**PDZ interaction between Neph2 and postsynaptic density (PSD)-95. (A)** Domain structure of Neph2 (778 aa long in rat). Ig, immunoglobulin domain; SP, signal peptide; TM, transmembrane domain; RRMQTHV*, the last 7 aa residues of Neph2 containing the type I PDZ-binding motif. **(B)** Yeast two-hybrid interactions between the C-termini of the Neph family proteins and the PDZ domains of PSD-95 family proteins (PSD-95, PSD-93/chapysin-110, SAP97 and SAP102). Underlined mutations (T776E and V778A) disrupt the PDZ interactions of Neph2. PDZ domains from ZO-1 and Shank1/GRIP1 were used as positive and negative controls, respectively. pBHA and pGAD10, bait and prey constructs, respectively. β-Galactosidase activity: +++, <45 min; ++, 45–90 min; +, 90–240 min; −, no significant β-gal activity. **(C)** Pull down of PSD-95 (full length) expressed in HEK293T cells by GST-Neph1/2/3 (last seven residues, WT or PDZ-binding mutants). GRIP2, a negative control. **(D)** Coimmunoprecipitation between Neph2 (full cytoplasmic region) and PSD-95 (full length) in HEK293T cells. Doubly transfected HEK cell lysates were immunoprecipitated with FLAG antibodies and immunoblotted. Two mutations that disrupt the PDZ interaction were used as negative controls (Δ3, last 3 aa deletion; TE, T776E). **(E)** PDZ interaction-dependent coclustering of Neph2 and PSD-95 in heterologous cells. COS-7 cells were transfected singly with Neph2 (full length) or PSD-95 (full length), or doubly with Neph2 (WT or C-terminally FLAG tagged) and PSD-95, and were immunostained as indicated. Scale bar, 10 μm.

We have identified Neph2 as a novel binding partner of PSD-95 through yeast two-hybrid screens. Neph2 contains five Ig-like domains in the extracellular region, followed by a single TM and a cytoplasmic region that ends with a type I PDZ domain-binding C-terminus (Figure 1A). The C-terminus of Neph2 interacted with the PDZ1 and PDZ2 domains (not PDZ3) of PSD-95, as supported by the effects of the point mutations in the Neph2 C-terminus (Figure 1B). Neph2 also interacted with other members of the PSD-95 family (PSD-93/chapsyn-110, SAP97 and SAP102). Two other Neph family proteins, Neph1 and Neph3, also interacted with PSD-95, although Neph3 exhibited a much weaker interaction.

The PDZ interaction between Neph2 and PSD-95 could also be confirmed by GST pull-down and *in vitro* coprecipitation assays (Figures 1C,D). When coexpressed in heterologous cells, Neph2 and PSD-95 were colocalized in discrete intracellular clusters in PDZ interaction-dependent manner, as shown by the loss of coclustering in the mutant Neph2 where the C-terminal PDZ-binding motif was blocked by FLAG tagging (Figure 1E). Singly expressed Neph2 displayed a similar localization to discrete intracellular clusters, whereas singly expressed PSD-95 was widespread throughout the cell, suggesting that Neph2 translocates PSD-95 to the clusters. Together, these results indicate that Neph2 directly interacts with PSD-95 through its C-terminal PDZ-binding motif.

### Expression Patterns of Neph2 Proteins

**Figure 2:**
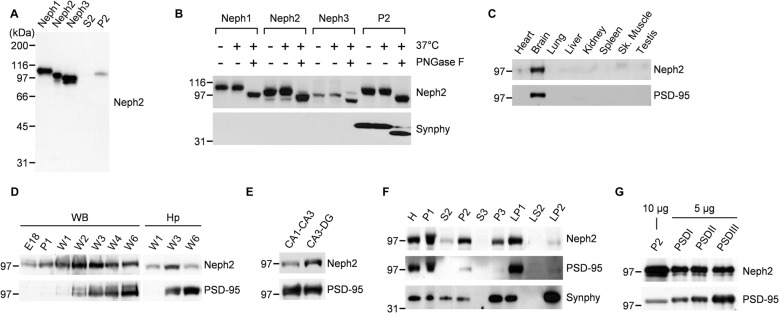
**Distribution patterns of Neph2 proteins in the brain. (A)** Neph2 antibodies recognize a single band in the rat brain that co-migrates with the Neph2 protein expressed in heterologous cells. Neph1/2/3 proteins expressed in HEK293T cells and the crude synaptosomal (P2) and extra-crude synaptosomal (S2) rat brain fractions (postnatal week 6) were immunoblotted with Neph2 antibodies. The numbers indicate molecular weights (kDa) of size markers used for Western blotting. **(B)** Neph2 in the brain is modified by N-glycosylation. Heterologously and brain-expressed Neph2 proteins were digested with PNGases and immunoblotted. Synaptophysin (Synphy) was used as a positive control. **(C)** Neph2 is highly expressed in the brain. Protein samples from the indicated rat tissues (postnatal week 6) were immunoblotted. Sk., skeletal. **(D)** Temporal expression of Neph2, reaching a peak postnatal week ~2–3 in both whole brain (WB) and hippocampus (Hp). E, embryonic; P, postnatal day; W, postnatal week. **(E)** Distribution of Neph2 proteins in hippocampal subregions (postnatal week 3). **(F)** Distribution of Neph2 proteins in rat brain subcellular fractions (postnatal week 6). H, Homogenates; P1, nuclei and other large debris; P2, crude synaptosomes; S2, supernatant after P2 precipitation; S3, cytosol; P3, light membranes; LP1, synaptosomal membranes; LS2, synaptosomal cytosol; LP2, synaptic vesicle-enriched fraction. **(G)** Distribution of Neph2 proteins in PSD fractions (postnatal week 6). Crude synaptosomal and PSD proteins were immunoblotted.

Previously our group (Choi et al., 2015) and Martin et al. (2015) independently reported *Neph2* expression in DG neurons using the GFP expression driven by the endogenous *Neph2* promoter in *Neph2^−/−^* mice (Prince et al., 2013). Martin et al. (2015) also found axonal and dendritic localization of the exogenous Neph2 proteins, and partial colocalization of the endogenous Neph2 proteins with excitatory synaptic markers (PSD-95 and vGlut1) in cultured hippocampal neurons. Based on the interaction between Neph2 and PSD-95 (Figure 1), we further investigated the expression patterns of Neph2 proteins. Our Neph2 polyclonal antibodies, directed against the last 10 aa residues of the protein, recognized all Neph proteins (Neph1, Neph2 and Neph3) expressed in heterologous cells to a similar extent, likely due to their similar aa sequences, but recognized only one band in the rat brain, which co-migrated with the Neph2 protein expressed in heterologous cells (Figure 2A). This *in vivo* band likely represents an authentic Neph2 because it is undetectable in Neph2-deficient (*Neph2^−/−^*) brains (Choi et al., 2015), and because the expression levels of *Neph1* and *Neph3* in mouse brain are much lower than that of *Neph2* from the *in situ* hybridization experiments performed by our group (Supplementary Figure S1) and Martin et al. (2015). Neph2 appears to be modified by N-glycosylation, as supported by a positive PNGase digestion (Figure 2B).

In immunoblot analysis, Neph2 proteins were most abundant in the brain relative to other tissues (Figure 2C). Neph2 protein levels gradually increased and peaked at postnatal week ~2–3 both in the whole brain (WB) and hippocampus, but gradually declined to adult levels in following weeks (Figure 2D). In the subregions of hippocampus, Neph2 proteins were more abundant in CA3-DG compared to CA1-CA3 (Figure 2E), consistent with the GFP expression pattern in *Neph2^−/−^* mice (Choi et al., 2015; Martin et al., 2015). In subcellular brain fractions, Neph2 proteins were more abundant in crude synaptosomal and synaptic membrane fractions (Figure 2F), and detected in PSD fractions (Figure 2G), in support of its excitatory postsynaptic localization.

### *In Vivo* Interaction between Neph2 and PSD-95

**Figure 3:**
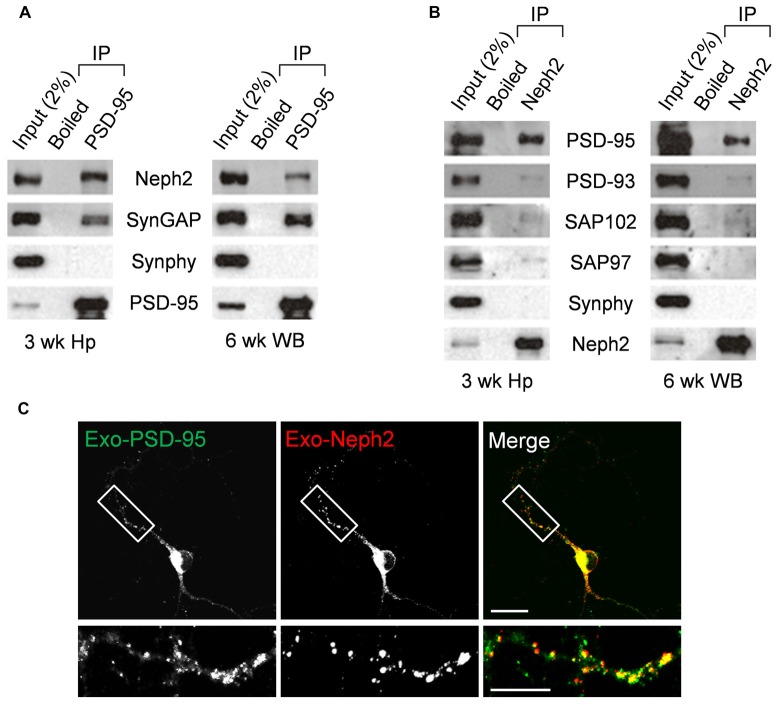
***In vivo* interaction between Neph2 and PSD-95. (A,B)** *In vivo* coimmunoprecipitation between Neph2 and PSD-95 family proteins in rat brains. Hippocampal (Hp) and WB lysates were immunoprecipitated with PSD-95, Neph2, or boiled Neph2/PSD-95 (control) antibodies and immunoblotted as indicated. Immunoblot for SynGAP (150 kDa), a known PSD-95-binding protein, was a positive control. Synphy (38 kDa), synaptophysin; wk, postnatal week. The molecular weights of PSD-93, SAP102 and SAP97 proteins are 110, 90 and 97 kDa, respectively. **(C)** Neph2 colocalizes with PSD-95 in cultured dentate granule (DG) neurons. Cultured DG neurons were transfected with Neph2 and PSD-95 at 8 days *in vitro* (DIV 8) and immunostained at DIV 10. Scale bar, up 20 μm, bottom 10 μm.

We next tested whether Neph2 and PSD-95 form a complex *in vivo* by performing coimmunoprecipitation experiments with rat brain synaptosomal lysates. In synaptosomal lysates of the hippocampus, PSD-95 coprecipitated with Neph2 as well as with SynGAP (a known PSD-95-binding protein; Figure 3A). A similar result was obtained from WB lysates. In a reverse-orientation experiment, Neph2 coprecipitated with PSD-95 and, to a much lesser extent, with PSD-95 relatives (PSD-93, SAP102 and SAP97) in both hippocampal and WB lysates (Figure 3B). Because the Neph2 antibody that we generated did not recognize endogenous Neph2 proteins under immunohistochemistry conditions, we exogenously expressed Neph2 and PSD-95 in cultured DG neurons and found that they were highly colocalized (Figure 3C). Together, these results indicate that Neph2 proteins interact with PSD-95 *in vivo*.

### Increased mEPSC Frequency of DG Neurons in *Neph2^−/−^* Mice at Postnatal Week 2 but Not at 3

Given the excitatory postsynaptic localization of Neph2 and its interaction with PSD-95, we investigated whether the excitatory synaptic transmission was altered in the DG neurons of *Neph2^−/−^* mice. Notably, Martin et al. (2015) reported normal frequency and amplitude of mEPSCs of *Neph2^−/−^* DG neurons at P17–21. Nevertheless, we noticed that Neph2 protein expression peaks from postnatal week 2 (Figure 2D). Therefore, we dissected the juvenile stages into postnatal week 2 (P14–17) and 3 (P20–22) and measured mEPSCs from *Neph2^−/−^* DG neurons in both male and female mice.

**Figure 4:**
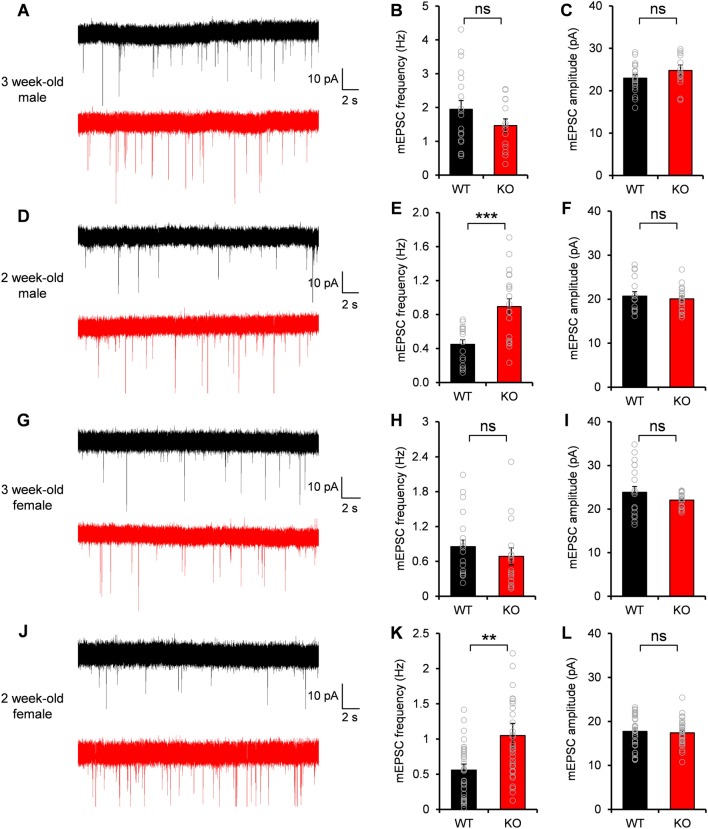
**Increased miniature excitatory postsynaptic current (mEPSC) frequency in *Neph2^−/−^* DG neurons at postnatal week 2 but not at 3. (A–C)** Normal frequency and amplitude of mEPSCs in DG neurons of male *Neph2^−/−^* mice at postnatal week 3 (*n* = 19 cells from 4 mice for WT, and 14, 4 for KO, ns, not significant, Student’s *t*-test). **(D–F)** Increased frequency but not amplitude of mEPSCs in DG neurons of male *Neph2^−/−^* mice at postnatal week 2 (*n* = 16 cells from 4 mice for WT, and 20, 4 for KO). **(G–I)** Normal frequency and amplitude of mEPSCs in DG neurons of female *Neph2^−/−^* mice at postnatal week 3 (*n* = 17 cells from 4 mice for WT, and 16, 4 for KO). **(J–L)** Increased frequency but not amplitude of mEPSCs in DG neurons of female *Neph2^−/−^* mice at postnatal week 2 (*n* = 34 cells from 9 mice for WT, and 33, 10 for KO). All data are presented as mean ± SEM. **P < 0.01; ***P < 0.001.

We found that the frequency and amplitude of mEPSCs were normal in DG neurons of male *Neph2^−/−^* mice at postnatal week 3 (Figures 4A–C), consistent with the previous report (Martin et al., 2015). At postnatal week 2, however, the frequency but not amplitude of mEPSCs was increased by ~2 folds in DG neurons of male *Neph2^−/−^* mice compared to WT mice (Figures 4D–F). We found the same results from female *Neph2^−/−^* mice; increased frequency of mEPSCs at postnatal week 2 but not at 3 (Figures 4G–L). These results indicate that *Neph2* deletion leads to increased excitatory synaptic transmission in DG neurons at postnatal week 2 but not at 3.

### Normal Evoked Excitatory Synaptic Transmission and Synaptic Plasticity of MPP-DG Synapses in *Neph2^−/−^* Mice at Postnatal Week 3

It was unexpected that the frequency of mEPSCs of *Neph2^−/−^* DG neurons was increased specifically at postnatal week 2, but not at 3. Despite of the normal mEPSCs, we suspected that other synaptic properties, such as evoked synaptic transmission and synaptic plasticity, might be altered in *Neph2^−/−^* DG neurons at postnatal week 3, as a consequence of synaptic connectivity defects at postnatal week 2.

**Figure 5:**
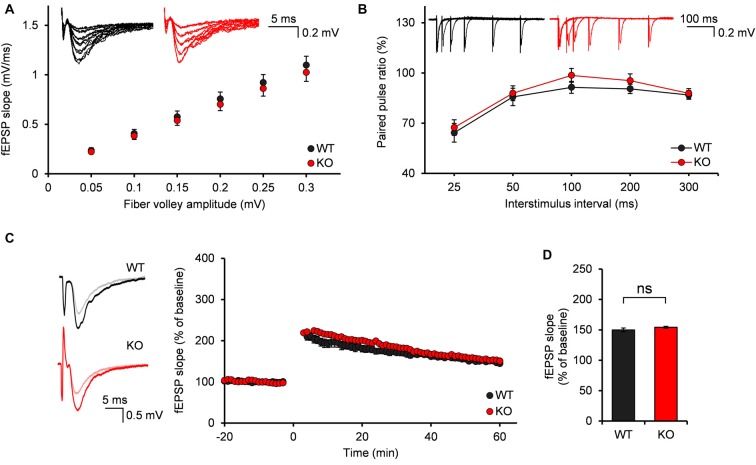
**Normal input-output ratio, paired-pulse ratio and synaptic plasticity of *Neph2^−/−^* medial perforant pathway (MPP)-DG synapses at postnatal week 3. (A)** Normal input-output curve, determined by plotting field excitatory postsynaptic potential (fEPSP) slopes against fiber volley amplitudes at MPP-DG synapses (P21–23; *n* = 11 slices from 5 mice for WT, and 14, 4 for KO, ns, not significant, Student’s *t*-test). **(B)** Normal paired-pulse ratio, determined by plotting the ratio of two consecutive field EPSP slopes plotted against inter-stimulus intervals at MPP-DG synapses (P21–23; *n* = 11, 5 for WT, and 14, 4 for KO). **(C)** Normal long-term potentiation (LTP) induced by theta burst stimulation (TBS) at MPP-DG synapses (P21–25; *n* = 12, 5 for WT, and 13, 5 for KO). **(D)** Quantification of the results in **(C)**, as shown by the magnitude of fEPSP slope (50–60 min). All data are presented as mean ± SEM.

We first investigated the evoked excitatory synaptic transmission at *Neph2^−/−^* medial perforant pathway (MPP)-DG synapses, as measured by plotting fEPSP slopes against fiber volley amplitudes (input-output ratio). We found that there was no significant difference between WT and *Neph2^−/−^* MPP-DG synapses (Figure 5A). In addition, the paired-pulse ratio was not different between genotypes (Figure 5B), suggesting that the presynaptic release probability is normal. Lastly, LTP induced by TBS at MPP-DG synapses was not different between genotypes (Figures 5C,D). Together, these results indicate that evoked synaptic transmission and synaptic plasticity are normal in *Neph2^−/−^* MPP-DG synapses at postnatal week 3.

## Discussion

In this study, we show some evidence indicating the postsynaptic localization of Neph2 proteins in the mammalian brain. Neph2 directly interacts with PSD-95 both *in vitro* and *in vivo*, and Neph2 proteins are detected in the brain PSD fraction. Moreover, Neph2 and PSD-95 are colocalized when coexpressed in cultured neurons. PSD-95, as an abundant excitatory postsynaptic scaffolding protein, interacts with many types of synaptic adhesion molecules and regulates their synaptic localization, clustering and coupling with signaling molecules (Han and Kim, 2008). Synaptic adhesion molecules could also recruit PSD-95 and other synaptic scaffolding proteins during synapse formation and development (Dalva et al., 2007; Missler et al., 2012). It was previously shown that Neph2 interacts with CASK, a presynaptic scaffolding protein, through its cytoplasmic region (Gerke et al., 2006; Bhalla et al., 2008). Whether these Neph2-PSD-95 and Neph2-CASK complexes exist at the post- and pre-side of the same synapse is unknown. However, considering the homophilic interaction of Neph2 through its extracellular domains (Martin et al., 2015), it is conceivable that Neph2 might be involved in *trans-synaptic* signaling via the scaffolding proteins and associated molecules.

Functionally, we found that the frequency but not the amplitude of mEPSCs was increased by ~2 folds in DG neurons of *Neph2^−/−^* mice at postnatal week 2. Increased frequency of mEPSCs could be due to increased presynaptic release probability and/or increased number of excitatory synapses, which is not clear at this moment. In either case, nevertheless, it is likely that Neph2 could be involved in negative regulation of synaptic development and function in the early postnatal developmental stage. Recent studies on synaptic adhesion molecules have suggested several novel mechanisms by which neuronal synapses can be negatively regulated. MDGA1 has been shown to interact with neuroligin 2 in a *cis* manner and inhibit neuroligin 2-dependent induction of presynaptic differentiation in contacting axons (Lee et al., 2013; Pettem et al., 2013). Semaphorin 5A negatively regulates dendritic spines and excitatory synapses in the DG neurons through mechanisms involving its receptor PlexinA2 and its cytoplasmic RasGAP domain (Duan et al., 2014). The Nogo receptor 1 (NgR1) restricts postsynaptic synapse formation in the hippocampus through its coreceptor TROY and the small GTPase RhoA (Wills et al., 2012). SALM4 inhibits the *trans*-synaptic SALM3 interaction with presynaptic LAR, and thereby suppressing SALM3-dependent presynaptic differentiation at excitatory synapses (Lie et al., 2016). Whether Neph2 inhibits excitatory synapses through similar mechanisms remains to be determined. Notably, SYG-1, the Kirrel ortholog in *Caenorhabditis elegans*, interacts with SKR-1 of Skp1-cullin-F-box (SCF) E3 ubiquitin ligase complex to regulate selective synapse elimination (Ding et al., 2007), and interacts with a key regulator of actin cytoskeleton, WAVE regulatory complex, to control synapse formation and axon branching (Chia et al., 2014). Whether these interactions are conserved for Neph2 proteins and whether they have similar roles in regulating synapses of the mammalian brain need to be investigated.

The increased frequency of mEPSCs in DG neurons of *Neph2^−/−^* mice is not observed at postnatal week 3. Moreover, the input-output ratio, paired-pulse ratio and synaptic plasticity of *Neph2^−/−^* MPP-DG synapses are normal at postnatal week 3. Previously, we also found that the frequency and amplitude of mEPSCs in DG neurons of *Neph2^−/−^* mice are normal at postnatal week 8 (Choi et al., 2015). Therefore, the synaptic connectivity defects in DG neurons of *Neph2^−/−^* mice could be specific during a narrow postnatal period when endogenous Neph2 protein expression peaks. As Neph2 is expressed in other brain regions including the cortex, striatum, olfactory bulb, and cerebellum, it would be interesting future direction to study whether the synapses of these brain regions of *Neph2^−/−^* mice also show age-specific connectivity defects.

It is notable that, similar to *Neph2^−/−^* mice, a mouse model of *Syngap1* haploinsufficiency shows age-specific synaptic defects in DG neurons (Clement et al., 2012). The frequency and amplitude of mEPSCs in DG neurons are increased at postnatal week 2, but normalized at postnatal week 3, in *Syngap1* heterozygous mice. As SynGAP1, a Ras GTPase-activating protein, is abundantly expressed in the PSD and also interacts with PSD-95 (Kim et al., 1998), it is possible that Neph2 and SynGAP1 form a protein complex through PSD-95 to tightly control synaptic development and function of DG neurons. Importantly, *SYNGAP1* haploinsufficiency has been strongly associated with intellectual disability and autism spectrum disorders (Hamdan et al., 2009, 2011), suggesting that synaptic defects of DG neurons during early postnatal period might be a common contributing risk factor for *NEPH2* and *SYNGAP1* related brain disorders.

Behaviorally, adult (postnatal week 8–16) *Neph2^−/−^* mice display moderate hyperactivity in a familiar environment and defective novel object preference (Choi et al., 2015). It is not easy at this moment to connect the synaptic connectivity defects in DG neurons of the early postnatal period with the behavioral changes in adult *Neph2^−/−^* mice. More comprehensive analysis on the behaviors of *Neph2^−/−^* mice in both juvenile and adult stages will help us better understand the causal link between synaptic defects and behavioral abnormalities of the mice.

In conclusion, our study identifies Neph2 as a novel binding partner of PSD-95 and suggests its potential role as a negative regulator of excitatory synaptic transmission in DG neurons during early postnatal period, which might be implicated in some neurodevelopmental and cognitive disorders associated with *NEPH2/KIRREL3* mutations.

## Author Contributions

JDR, Su-YC, YSC, T-YC, J-SP, WC, HP, DL, M-HK, YL, J-SR and KH designed and performed the experiments. JDR, Su-YC, YSC, T-YC, J-SP, J-SR, HK, JK, Se-YC and YCB analyzed and interpreted the data. TC generated *Neph2^−/−^* mice. KS, KH and EK supervised the project and wrote the article. All authors read and approved the manuscript.

## Funding

This study was supported by the National Research Foundation of Korea (NRF) grant funded by the Korea government Ministry of Science, ICT and Future Planning (MISP; NRF-2015R1C1A1A01052794 to KH) and (NRF-2012M3A9B6055378 to HK), the grant of the Korea Health Technology R&D Project through the Korea Health Industry Development Institute (KHIDI) funded by the Ministry of Health and Welfare, South Korea (HI16C0090 to KH) and the Institute for Basic Science (IBS-R002-D1 to EK).

## Conflict of Interest Statement

The authors declare that the research was conducted in the absence of any commercial or financial relationships that could be construed as a potential conflict of interest.

## Supplementary Material

FIGURE S1 **Distribution of Neph1/2/3/mRNAs in the mouse brain revealed by *in situ* hybridization.** Mouse brain sections (6 weeks) were hybridized with specific Neph1/2/3 riboprobes. Neph2 mRNA is highly expressed in DG neurons. The expression levels of Neph1/3 mRNAs are much less than that of Neph2 mRNA.
