# Supplementary figures and images for: Increased Excitatory Synaptic Transmission of Dentate Granule Neurons in Mice Lacking PSD-95-Interacting Adhesion Molecule Neph2/Kirrel3 during the Early Postnatal Period
Source: Front Mol Neurosci. 2017 Mar 22;10:81. doi: 10.3389/fnmol.2017.00081 (PMC5360738; doi:10.3389/fnmol.2017.00081)

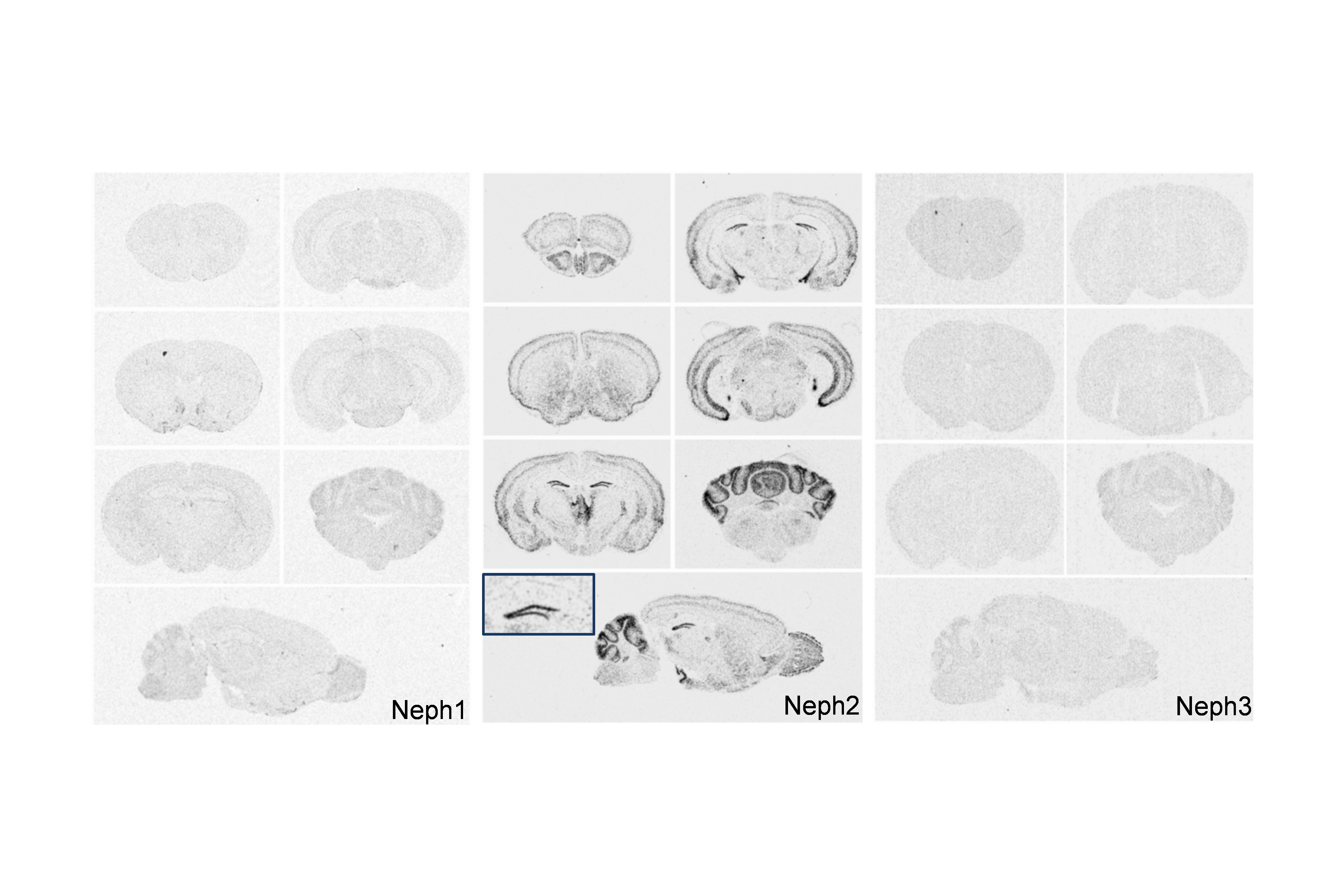

Supplement: FIGURE S1 — Distribution of Neph1/2/3/mRNAs in the mouse brain revealed by in situ hybridization. Mouse brain sections (6 weeks) were hybridized with specific Neph1/2/3 riboprobes. Neph2 mRNA is highly expressed in DG neurons. The expression levels of Neph1/3 mRNAs are much less than that of Neph2 mRNA. [file Image_1.jpeg]
